# Clinicopathological Features and Prognostic Impact of Human Epidermal Growth Factor Receptor 2-Low Status in Early-Stage Breast Cancer: A Single-Center Cohort Hormone Receptor-Stratified Retrospective Analysis

**DOI:** 10.7759/cureus.114911

**Published:** 2026-08-21

**Authors:** Mai A Abdelazez, Ahmed M. Hammad, Bashayer Alenazi, Laila Moharram, Omar Alzahrani

**Affiliations:** 1 Medical Oncology Department, Prince Sultan Oncology Center, King Salman Armed Forces Hospitals in Northwest Region, Tabuk, SAU; 2 Clinical Oncology and Nuclear Medicine Department, Faculty of Medicine, Aswan University, Aswan, EGY; 3 Pathology Department, King Salman Armed Forces Hospitals in Northwest Region, Tabuk, SAU

**Keywords:** breast cancer, her2-low, hormone receptor status, middle eastern population, prognosis, survival outcomes

## Abstract

Introduction

This study evaluates the prevalence, clinicopathological features, and prognostic impact of human epidermal growth factor receptor 2-low (HER2-low) on breast cancer (BC), focusing on hormone receptor (HR)-stratified outcomes.

Materials and methods

We retrospectively analyzed 97 patients with HER2-negative invasive BC from 2017 to 2024 at a single center. Data were compared between HER2-low and HER2-0 cohorts, stratified by HR status. Survival outcomes, including disease-free survival (DFS), metastasis-free survival (MFS), and overall survival (OS), were estimated using Kaplan-Meier methodology. Univariate and multivariate Cox regression analyses were performed to evaluate the prognostic effect of HER2-low on DFS.

Results

The prevalence of HER2-low was 61.9%, with 83.3% of our cases being hormone receptor-positive (HR+). HER2-low/HR+ tumors showed significant disparities in clinical T-stage (p=0.029) compared to HER2-0/HR+ cases. In the hormone receptor-negative (HR-) subgroup, HER2-low tumors exhibited significantly higher lymphovascular invasion (p=0.035) and high cellular proliferation, with all cases having Ki-67 > 20%. In our cohort, lymph node ratio (LNR) risk category and Ki-67 index were identified as the primary independent predictors of DFS. While HER2-low status showed a trend toward increased hazard in univariate analysis, it did not maintain independent significance in the multivariable model (p=0.217). Notably, subgroup analysis demonstrated that the protective effect of HR positivity was significantly more robust within the HER2-low population (p=0.008) compared to the HER2-0 group (p=0.221).

Conclusions

HER2-low BC is highly prevalent in our cohort but does not independently influence early-stage prognosis. Instead, underlying tumor biology, specifically proliferation (Ki-67) and nodal burden (LNR), remains the primary driver of clinical outcomes. Hormone receptor positivity was associated with favorable survival within the HER2-low subgroup, although non-significant interaction testing underscores that these subgroup observations are exploratory.

## Introduction

Breast cancer (BC) is a diverse malignancy characterized by a complicated relationship between genomic structure, transcriptional signals, patterns of protein expression, and the tumor microenvironment, all of which affect therapeutic and clinical outcomes [1]. Human epidermal growth factor receptor 2 (HER2) has been used as a prognostic and predictive biomarker in BC, with standardized immunohistochemistry and in situ hybridization criteria sorting the disease into HER2-positive and HER2-negative groups. Although frequently used in clinical decision-making, this binary framework is now regarded as oversimplified, since emerging evidence indicates that HER2 expression occurs across a biological spectrum rather than being restricted to a strict positive-negative distinction [2,3]. Within this spectrum, the clinically important subtype known as “HER2-low” emerges, which is characterized by immunohistochemistry (IHC) 1+ or IHC 2+ staining in the absence of Erb-B2 receptor tyrosine kinase 2 (ERBB2) gene amplification.

Several research studies reported that HER2-low malignancies exhibit intermediate levels of ERBB2 expression, unique transcriptional signatures, altered PI3K/AKT and MAPK pathway activation, and context-specific interaction with estrogen receptor signaling in luminal tumors [4-6]. These features differentiate HER2-low tumors biologically from HER2-0, despite both historically falling under the “HER2-negative” term.

Worldwide prevalence data highlight the magnitude of this phenotype. Across large real-world cohorts, HER2-low tumors account for 45%-65% of all breast cancers, with even higher prevalence in hormone receptor-positive disease, frequently exceeding 70% [7,8]. Geographic variability leads to additional complexity: East Asian cohorts commonly report some of the highest HER2-low rates globally, whereas European and North American populations reveal slightly lower proportions [9,10]. These differences mostly reflect a combination of intrinsic molecular variation and substantial variability in HER2 scoring reproducibility. In fact, discriminating IHC 0 from IHC 1+ remains one of the least consistent areas in breast pathology. The subtlety of faint, incomplete membranous staining and the effect of pre-analytic and analytic factors such as fixation, antibody clone selection, and staining platform heterogeneity result in a frequently poor interobserver agreement, even among expert breast pathologists [11,12].

The clinical relevance of HER2-low disease escalated dramatically following the success of antibody-drug conjugates (ADCs), most notably trastuzumab deruxtecan (T-DXd). Unlike traditional HER2-targeted therapies that require high HER2 receptor density to exert activity, T-DXd couples a high-affinity anti-HER2 antibody with a potent topoisomerase-I payload via a cleavable linker that enables a bystander effect, allowing cytotoxic diffusion into adjacent tumor cells regardless of HER2 expression uniformity [13,14]. The landmark DESTINY-Breast04 trial demonstrated that patients with HER2-low metastatic breast cancer significantly obtain progression-free and overall survival from T-DXd compared with standard chemotherapy, firmly positioning HER2-low as a predictive biomarker for ADC sensitivity rather than merely a pathological descriptor [13].

Despite therapeutic progress, the prognostic significance of HER2-low expression in early breast cancer remains unsettled. Some retrospective cohorts initially reported more favorable clinicopathological features, particularly within hormone receptor-positive populations, such as smaller size, lower grade, reduced proliferation, and lower lymphovascular invasion [15,16]. However, robust analyses and meta-studies consistently demonstrate that HER2-low expression is not independently prognostic once hormone receptor biology, tumor grade, and intrinsic subtype characteristics are taken into consideration [17].

Many studies show overlapping survival outcomes for HER2-low and HER2-0 tumors once stratified by hormone receptor (HR) status, suggesting previous observations of improved prognosis were influenced by luminal enrichment within the HER2-low category [3,18]. While HR-negative/HER2-low tumor clusters tend to be more aggressive and closer to basal-like patterns, HR-positive/HER2-low tumors follow luminal transcriptional pathways and are enriched for ESR1 and PIK3CA alterations [19,20].

Accordingly, we conducted a real-world study of patients with early breast cancer treated at a tertiary oncology center in the Middle East to clarify the prevalence, clinicopathological features, and prognostic relevance of HER2-low disease compared with HER2-0 tumors. Given evidence that hormone receptor status fundamentally modulates HER2-low biology, we performed HR-stratified analyses to determine whether HR-negative/HER2-low cancers behave distinctly from HR-positive/HER2-low disease. By integrating detailed pathological features and survival outcomes, this study aims to address key knowledge gaps and provide robust regional insights at a time when HER2-low classification is transitioning from a descriptive category to a biomarker with direct therapeutic implications in the ADC era [21].

## Materials and methods

Study design

We conducted a retrospective analysis of a cohort of patients diagnosed with early BC using data from a well-maintained institutional database (electronic medical records, pathology reports, radiology reports, and other relevant documents) at a tertiary oncology center in Northwest Saudi Arabia from January 2017 to October 2024. Inclusion criteria covered all female patients who were 18 years of age or older at diagnosis, had a histologically proven primary invasive localized HER2-negative breast cancer (stage I-III), and were operated on in our institution. Patients were excluded if they had incomplete diagnostic data, HER2-positive status, unknown HER2 status, carcinoma in situ, or stage IV (M1) disease. This study provides clinical insights into HER2-low BC features in the Middle Eastern population, which is currently under-represented in the global HER2-low literature.

Data collection

Detailed clinicopathological data were collected for all patients, encompassing demographic details such as age and body mass index (BMI), in addition to menopausal status, and clinical assessment included tumor location, size (cT), and nodal status (cN) according to the eighth edition of the American Joint Committee on Cancer (AJCC) clinical tumor-node-metastasis (TNM) staging system. Furthermore, the dataset included surgical approach (breast and axillary), histopathological characteristics (type, grade, lymphovascular invasion (LVI), and ductal carcinoma in situ (DCIS) presence), and the lymph node ratio (LNR) risk categories. LNR was calculated using the following formula: (number of positive lymph nodes ÷ total number of lymph nodes removed). Following established prognostic models [22], LNR categories were grouped into commonly used risk categories (Table 1). Neoadjuvant and different adjuvant treatment modalities (chemotherapy, radiotherapy, and endocrine therapy) were documented. Chemotherapy timing was categorized into three groups: no chemotherapy, adjuvant chemotherapy, and neoadjuvant chemotherapy. In the Cox proportional hazards models, “no chemotherapy” was used as the reference group to evaluate the relative impact of treatment timing on disease-free survival (DFS). The patients were followed until October 30, 2025.

**Table 1:** LNR risk categories LNR: lymph node ratio

Risk category	LNR value
Low risk	≤0.20
Intermediate risk	0.21-0.65
High risk	>0.65

Molecular profiling and IHC

HR status, HER2 expression, and Ki-67 levels were documented. For IHC, ER (VENTANA clone SP1), PR (Bio SB, clone RBT-22), and HER2 (Ventana clone 4B5) were performed on 10% buffered formalin-fixed paraffin-embedded sections using the Ventana Ultra-View DAB Detection Kit on a Ventana BenchMark Ultra platform.

HER2-low status was defined according to American Society of Clinical Oncology (ASCO)/College of American Pathologists (CAP) guidelines as immunohistochemistry (IHC) 1+ or IHC 2+ with negative in situ hybridization (ISH-), while HER2-0 was defined as IHC 0.

It is a routine in our center that all breast cancer cases get reviewed by two pathologists (this includes ER, PR, and HER2 interpretation). After the introduction of the HER2-low concept, this double review continued with more scrutiny for the distinction between Her2 score 0 and 1+.

Sample size

The minimum required sample size was calculated as 97 participants, assuming a 50% anticipated population proportion and a 10% margin of error at a 95% confidence level (CI), using Sample Size 2.0 software. The 10% margin of error was selected considering the exploratory, single-center retrospective nature of the study. The sample size limits statistical power for subgroup survival analysis, which should be regarded as exploratory.

End points

The primary end point was to identify the main clinicopathological differences between HER2-0 and HER2-low early breast cancer. Secondary end points included overall survival (OS), which was defined as the time from the date of surgery to the date of death from any cause, and disease-free survival (DFS), which was defined as the time from the date of surgery to the first occurrence of either a local or distant breast cancer recurrence or death from breast cancer. Metastasis-free survival (MFS) was defined as the time from surgery to a distant recurrence or death from breast cancer. The whole population was divided into four groups based on HR and HER2 status.

Statistical method

Frequencies and percentages were used to convey descriptive statistics, particularly to identify the prevalence and what characteristics HER2-low tumors have in the group of patients with early-stage breast cancer. In inferential statistics, categorical data were evaluated using chi-square tests or Fisher’s exact tests. Survival outcomes (OS, DFS, and MFS) were estimated using Kaplan-Meier curves and compared via the log-rank test. Cox regression with univariate and multivariate analysis to evaluate prognostic factors affecting DFS was conducted. The statistical analyses were performed using SPSS version 26 (IBM Corp., Armonk, NY), and a significance threshold was set at p<0.05. Cases with missing primary end point or critical clinicopathological variables were excluded from multivariate models; complete case analysis was performed. Diagnostic interobserver agreement for HER2 scoring was maintained via routine independent dual-pathologist review, with consensus reaching 100% prior to statistical analysis.

## Results

General clinicopathological characteristics

A total of 97 female patients with primary invasive breast cancer were included. HER2-low status was identified in 61.9% of the cohort, while 38.1% were categorized as HER2-0 (Table 2). The prevalence of HER2-low was markedly greater in hormone receptor-positive (HR+) tumors compared to triple-negative breast cancer (TNBC) (83.3% versus 16.7%).

**Table 2:** Demographic and clinical features of the study population Data are presented as frequencies and percentages (number (%)). HER2: human epidermal growth factor receptor 2, BMI: body mass index, BCS: breast-conserving surgery, TM: total mastectomy, SLNB: sentinel lymph node biopsy, ALND: axillary lymph node dissection, HR: hormone receptor, TNM: tumor, node, metastasis classification, DCIS: ductal carcinoma in situ, LVI: lymphovascular invasion, IDC: invasive ductal carcinoma, ILC: invasive lobular carcinoma

Feature	Variables	Frequency (N=97)	Percent (%)
HER2 status	HER2 0	37	38.10%
	HER2-low	60	61.90%
Age (years)	>50	55	56.70%
	≤50	42	43.30%
Menopausal status	Postmenopausal	52	53.60%
	Premenopausal	45	46.40%
BMI (kg/m^2^)	>25	69	71.10%
	≤25	28	28.90%
Tumor location	Right	55	56.70%
	Left	39	40.20%
	Bilateral	3	3.10%
Breast surgery	BCS	44	45.40%
	TM	53	54.60%
Axillary surgery	SLNB	18	18.60%
	ALND	79	81.40%
Histological grade	I	3	3.10%
	II	44	45.40%
	III	50	51.50%
HER2 score	0	37	38.10%
	1+	47	48.50%
	2+	13	13.40%
HR status	HR-positive	76	78.40%
	HR-negative	21	21.60%
Clinical T-stage	cT1	25	25.80%
	cT2	51	52.60%
	cT3	15	15.50%
	cT4	6	6.20%
Clinical N-stage	cN0	51	52.60%
	cN1	28	28.90%
	cN2	17	17.50%
	cN3	1	1.00%
Clinical TNM stage	cIA	14	14.40%
	cIB	13	13.40%
	cIIA	34	35.10%
	cIIB	14	14.40%
	cIIIA	9	9.30%
	cIIIB	8	8.20%
	cIIIC	5	5.20%
DCIS component	Negative/absent	46	47.40%
	Low	14	14.40%
	Intermediate	15	15.50%
	High	22	22.70%
LVI	LVI-negative	86	88.70%
	LVI-positive	11	11.30%
Ki-67 index	≥20	61	62.90%
	<20	36	37.10%
Pathological subtype	IDC	74	76.30%
	ILC	12	12.40%
	Tubular	3	3.10%
	Mucinous	1	1.00%
	Medullary	1	1.00%
	Papillary	1	1.00%
	Others	5	5.20%
Adjuvant radiotherapy	Yes	83	85.60%
	No	11	11.30%
	Unknown	3	3.10%
Chemotherapy timing	Adjuvant	40	41.20%
	Neoadjuvant	44	45.40%
	None	13	13.40%
Adjuvant endocrine therapy	Yes	72	74.20%
	No	23	23.70%
	Unknown	2	2.10%

Patients in the HER2-low group received adjuvant hormonal therapy significantly more than those in the HER2-0 group (p=0.035). Regarding histological subtypes, there was a borderline significant difference (p=0.058), with an elevated proportion of invasive lobular carcinoma (ILC) in the HER2-low group (18.3%) compared to the HER2-0 group (2.7%). Both groups showed a comparable predominance of right-sided breast cancer (56.7% versus 56.8%), while all bilateral cases (5%) were exclusively in the HER2-low population (Table 3).

**Table 3:** Baseline clinicopathological characteristics of patients stratified by HER2 and HR status (N=97) Data presentation: Values are presented as numbers and percentages (%). Percentages may not total 100% due to rounding. Statistical analysis: p-values were calculated using Pearson’s chi-square test or Fisher’s exact test for categorical variables. Statistical significance was defined as p<0.05. HR: hormone receptor, HER2: human epidermal growth factor receptor 2, BMI: body mass index, TM: total mastectomy, BCS: breast-conserving surgery, ALND: axillary lymph node dissection, SLN: sentinel lymph node biopsy, TNM: tumor, node, metastasis staging system, LVI: lymphovascular invasion, DCIS: ductal carcinoma in situ, IDC: invasive ductal carcinoma, ILC: invasive lobular carcinoma

Features	Variable	All patients (N=97)			HR-negative (n=21) (21.6%)			HR-positive (n=76) (78.4%)		
		HER2-0 (n=37) (38.2%)	HER2-low (n=60) (61.8%)	p-value	HER2-0 (n=11) (52.4%)	HER2-low (n=10) (47.6%)	p-value	HER2-0 (n=26) (34.2%)	HER2-low (n=50) (65.8%)	p-value
Age	>50	20 (54.1%)	35 (58.3%)	0.679	4 (36.4%)	6 (60%)	0.279	16 (61.5%)	29 (58%)	0.766
	≤50	17 (45.9%)	25 (41.7%)		7 (63.6%)	4 (40%)		10 (38.5%)	21 (42%)	
Menopausal status	Premenopausal	19 (51.4%)	26 (43.3%)	0.442	8 (72.7%)	4 (40%)	0.198	11 (42.3%)	22 (44%)	0.888
	Postmenopausal	18 (48.6%)	34 (56.7%)		3 (27.3%)	6 (60%)		15 (57.7%)	28 (56%)	
BMI	>25	23 (62.2%)	46 (76.7%)	0.126	5 (45.5%)	7 (70%)	0.387	18 (69.2%)	39 (78%)	0.416
	≤25	14 (37.8%)	14 (23.3%)		6 (54.5%)	3 (30%)		8 (30.8%)	11 (22%)	
Location	Right	21 (56.8%)	34 (56.7%)	0.551	6 (54.5%)	4 (40%)	0.67	15 (57.7%)	30 (60%)	0.583
	Left	16 (43.2%)	23 (38.3%)		5 (45.5%)	6 (60%)		11 (42.3%)	17 (34%)	
	Bilateral	0 (0%)	3 (5%)		0 (0%)	0 (0%)		0 (0%)	3 (6%)	
Breast surgery	TM	16 (43.2%)	28 (46.7%)	0.742	6 (54.5%)	6 (60%)	1.00	10 (38.5%)	22 (44%)	0.807
	BCS	21 (56.8%)	32 (53.3%)		5 (45.5%)	4 (40%)		16 (61.5%)	28 (56%)	
Axillary surgery	ALND	29 (78.4%)	50 (83.3%)	0.542	6 (54.5%)	7 (70%)	0.659	23 (88.5%)	43 (86%)	1.00
	SLN	8 (21.6%)	10 (16.7%)		5 (45.5)	3 (30%)		3 (11.5%)	7 (14%)	
cT stage	cT1	10 (27%)	15 (25%)	0.116	2 (18.2%)	1 (10%)	1.00	8 (30.8%)	14 (28%)	0.029
	cT2	18 (48.6%)	33 (55%)		6 (54.5%)	7 (70%)		12 (46.2%)	26 (52%)	
	cT3	4 (10.8%)	11 (18.3%)		2 (18.2%)	1 (10%)		2 (7.7%)	10 (20%)	
	cT4	5 (13.5%)	1 (1.7%)		1 (9.1%)	1 (10%)		4 (15.4%)	0 (0%)	
cN stage	cN0	20 (54.1%)	31 (51.7%)	0.759	7 (63.6%)	5 (50%)	0.329	13 (50%)	26 (52%)	0.899
	cN1	9 (24.3%)	19 (31.7%)		2 (18.2%)	4 (40%)		7 (26.9%)	15 (30%)	
	cN2	8 (21.6%)	9 (15%)		2 (18.2%)	0 (0%)		6 (23.1%)	9 (18%)	
	cN3	0 (0%)	1 (1.7%)		0 (0%)	1 (10%)		0 (0%)	0 (0%)	
Clinical TNM stage	cIA	3 (8.1%)	11 (18.3%)	0.137	0 (0%)	0 (0%)	0.527	3 (11.5%)	11 (18.3%)	0.191
	cIB	8 (21.6%)	5 (8.3%)		2 (18.2%)	1 (10%)		6 (23.1%)	4 (8%)	
	cIIA	11 (29.7%)	23 (38.3%)		2 (18.2%)	0 (0%)		9 (34.6%)	23 (46%)	
	cIIB	4 (10.8%)	10 (16.7%)		2 (18.2%)	5 (50%)		2 (7.7%)	5 (10%)	
	cIIIA	3 (8.1%)	6 (10%)		0 (0%)	0 (0%)		3 (11.5%)	6 (12%)	
	cIIIB	4 (10.8%)	4 (4.9%)		3 (27.3%)	3 (30%)		1 (3.8%)	1 (2%)	
	cIIIC	4 (10.8%)	1 (1.7%)		2 (18.2%)	1 (10%)		2 (7.7%)	0 (0%)	
LVI	LVI-positive	4 (10.8%)	7 (11.7%)	1.00	0 (0%)	4 (40%)	0.035	4 (15.4%)	3 (6%)	0.222
	LVI-negative	33 (89.2%)	53 (88.3%)		11 (100%)	6 (60%)		22 (84.6%)	47 (94%)	
Ki-67	Ki-67 > 20%	25 (67.6%)	36 (60%)	0.454	9 (81.8%)	10 (100%)	0.476	16 (61.5%)	26 (52%)	0.474
	Ki-67 ≤ 20%	12 (32.4%)	24 (40%)		2 (18.2%)	0 (0%)		10 (38.5%)	24 (48%)	
Lymph node ratio risk categories	Low risk	27 (73%)	46 (76.7%)	0.682	10 (90.9%)	7 (70%)	0.311	17 (65.4%)	39 (78%)	0.278
	Intermediate and high risk	10 (27%)	14 (23.3%)		1 (9.1%)	3 (30%)		9 (34.6%)	11 (22%)	
Histological grade	I	0 (0%)	3 (5%)	0.26	0 (0%)	0 (0%)	1.00	0 (0%)	3 (6%)	0.407
	II	15 (40.5%)	29 (48.3%)		2 (18.2%)	1 (10%)		13 (50%)	28 (56%)	
	III	22 (59.5%)	28 (46.7%)		9 (81.8%)	9 (90%)		13 (50%)	19 (38%)	
DCIS	DCIS-negative	21 (56.8%)	25 (41.7%)	0.474	8 (72.7%)	6 (60%)	0.283	13 (50%)	19 (38%)	0.809
	Low	4 (10.8%)	10 (16.7%)		0 (0%)	2 (20%)		4 (15.4%)	8 (16%)	
	Intermediate	4 (10.8%)	11 (18.3%)		0 (0%)	1 (10%)		4 (15.4 %)	10 (20%)	
	High	8 (21.6%)	14 (23.3%)		3 (27.3%)	1 (10%)		5 (19.2%)	13 (26%)	
Pathological subtypes	IDC	30 (81.1%)	44 (73.3%)	0.058	10 (90%)	8 (80%)	0.586	20 (76.9%)	36 (72%)	0.109
	ILC	1 (2.7%)	11 (18.3%)		0 (0%)	1 (10%)		1 (3.8%)	10 (20%)	
	Mucinous	0 (0%)	1 (1.7%)		0 (0%)	0 (0%)		0 (0%)	1 (2%)	
	Others	3 (8.1%)	2 (3.1%)		1 (10%)	0 (0%)		2 (7.7%)	2 (4%)	
	Medullary	0 (0%)	1 (1.7%)		0 (0%)	1 (10%)		0 (0%)	0 (0%)	
	Papillary	1 (2.7%)	0 (0%)		0 (0%)	0 (0%)		1 (3.8%)	0 (0%)	
	Tubular	2 (5.4%)	1 (1.7%)		0 (0%)	0 (0%)		2 (7.7%)	1 (2%)	
Chemotherapy timing	Adjuvant	16 (43.2%)	24 (40%)	0.679	4 (36.4%)	5 (50%)	1.00	12 (46.2%)	19 (38%)	0.708
	Neoadjuvant	15 (40.5%)	29 (48.3%)		5 (45.5%)	4 (40%)		10 (38.4%)	25 (50%)	
	None	6 (16.2%)	7 (11.7%)		2 (18.2%)	1 (10%)		4 (15.4%)	6 (12%)	
Adjuvant radiotherapy	No	3 (8.1%)	8 (13.3%)	0.071	1 (9.1%)	2 (20%)	1.00	2 (7.7%)	6 (12%)	0.216
	Yes	31 (83.8%)	52 (86.7%)		9 (81.8%)	8 (80%)		22 (84.6%)	44 (88%)	
	Unknown	3 (8.1%)	0 (0%)		1 (9.1%)	0 (0%)		2 (7.7%)	0 (0%)	
Adjuvant hormonal therapy	No	12 (32.4%)	11 (18.3%)	0.035	9 (81.8%)	9 (90%)	1.00	3 (11.5%)	2 (4%)	0.142
	Yes	23 (62.2%)	49 (81.7%)		1 (9.1%)	1 (10%)		22 (84.6 %)	48 (96%)	
	Unknown	2 (5.4%)	0 (0%)		1 (9.1%)	0 (0%)		1 (3.8%)	0 (0%)	

Clinicopathological characteristics stratified by HR status

The majority of our population was HR+ (78.4%), with 21.6% being TNBC. In the HR+ subgroup, a significant difference was reported in clinical T-stage (p=0.029). Of note, all cT4 cases in the HR+ population were in the HER2-0 subgroup (15.4%). High-grade (grade III) tumors were more frequently found in HER2-0/HR+ compared to HER2-low/HR+ cases (50% versus 38%, respectively) (Table 3).

In the HR-negative (TNBC) subgroup, LVI positivity was significantly greater in HER2-low/HR- cases compared to HER2-0/HR- (40% versus 0%; p=0.035). Furthermore, all cases in the HER2-low/HR- subgroup expressed high cellular proliferation with a Ki-67 index > 20%. There were no significant differences among the groups for other characteristics such as age, BMI, menopausal status, and treatment modes (radiotherapy and chemotherapy) (Table 3).

Survival outcomes

The mean follow-up duration was 45.55 months (range: 11-100 months). For the entire cohort, the overall survival (OS) rate was 90.7% (95% CI: 81.69-99.79), disease-free survival (DFS) was 81.5% (95% CI: 73.96-89.10), and metastasis-free survival (MFS) was 83% (95% CI: 75.66-90.34) (Table 4).

**Table 4:** HER2 expression survival outcomes in matched patients Data presentation: Data are presented as mean percentages with 95% CI. Statistical analysis: p-values were determined using the log-rank test for survival rate comparisons. Statistical significance was defined as p<0.05. OS: overall survival, DFS: disease-free survival, MFS: metastasis-free survival, CI: confidence interval, HER2: human epidermal growth factor receptor 2

Survival outcome (95% CI)	HER2-0	HER2-low	p-value
OS	90.75 (81.70-99.79)	85.03 (76.99-93.06)	0.378
DFS	81.37 (68.50-94.25)	81.45 (72.15-90.75)	0.799
MFS	82.17 (69.25-95.09)	83.37 (74.57-92.17)	0.945

Survival rates according to HER2 status

**Figure 1:**
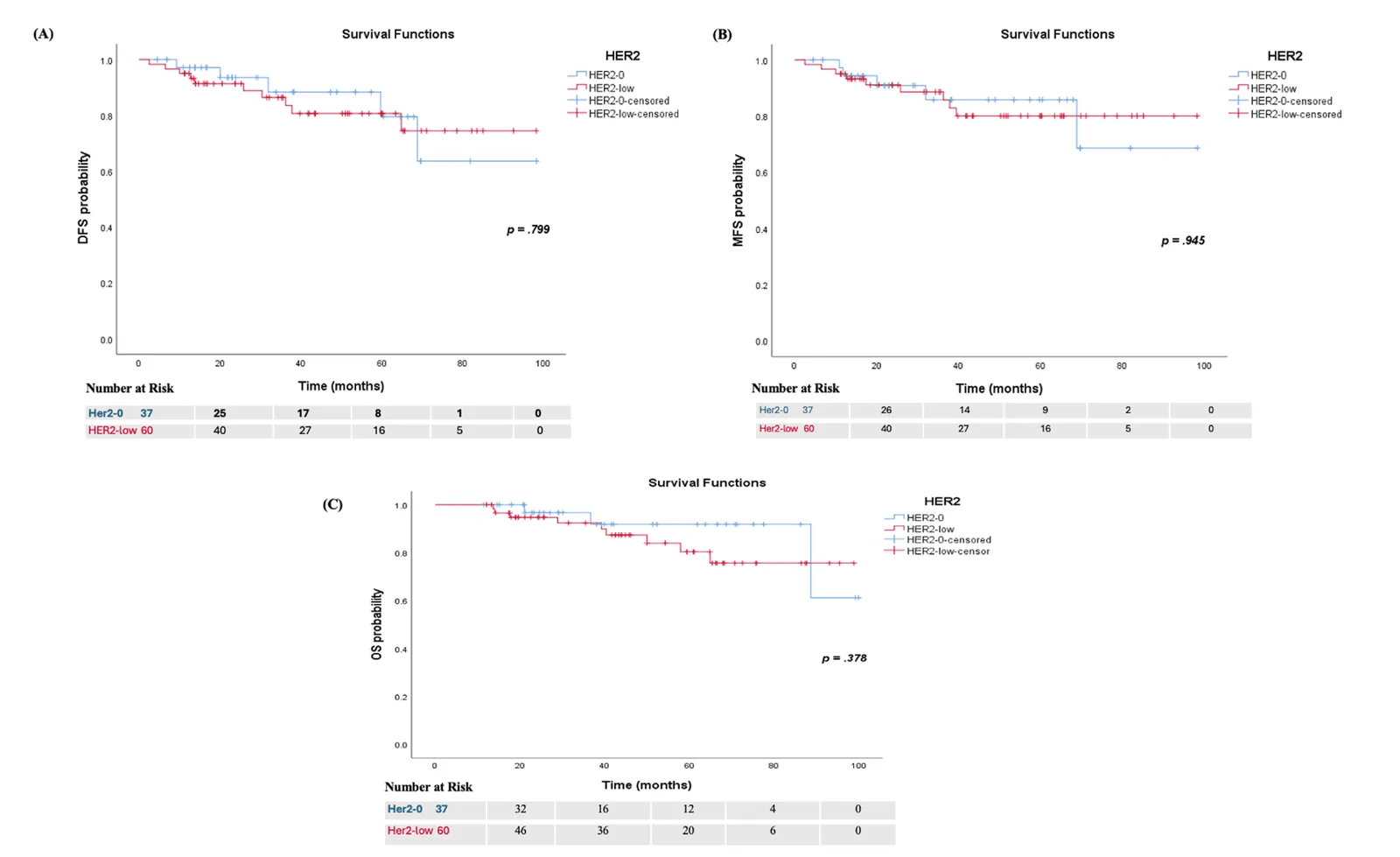
Survival outcomes of all patients based on HER2 status (A) Kaplan-Meier curve comparing DFS between HER2-low (n=60) and HER2-0 (n=37) groups. (B) Kaplan-Meier survival curve comparing MFS between HER2-low (n=60) and HER2-0 (n=37) groups. (C) Kaplan-Meier curve comparing OS between HER2-low (n=60) and HER2-0 (n=37) groups. p-values were calculated using the log-rank test. The number at risk is provided below the x-axis. HER2: human epidermal growth factor receptor 2, HR: hormone receptor, DFS: disease-free survival, MFS: metastasis-free survival, OS: overall survival

When comparing survival rates according to HER2 status, no significant differences were observed in OS, DFS, or MFS between the HER2-low and HER2-0 subtypes (Figure 1, Table 4).

Survival rates according to HR status

When analyzing the entire cohort regardless of HER2 status, HR expression was a significant determinant of recurrence-related outcomes. Patients with HR+ tumors exhibited significantly higher survival rates compared to those with HR- tumors (DFS: 87.9% versus 54.3% (p=0.003), MFS: 88.0% versus 64.7% (p=0.021)).

**Figure 2:**
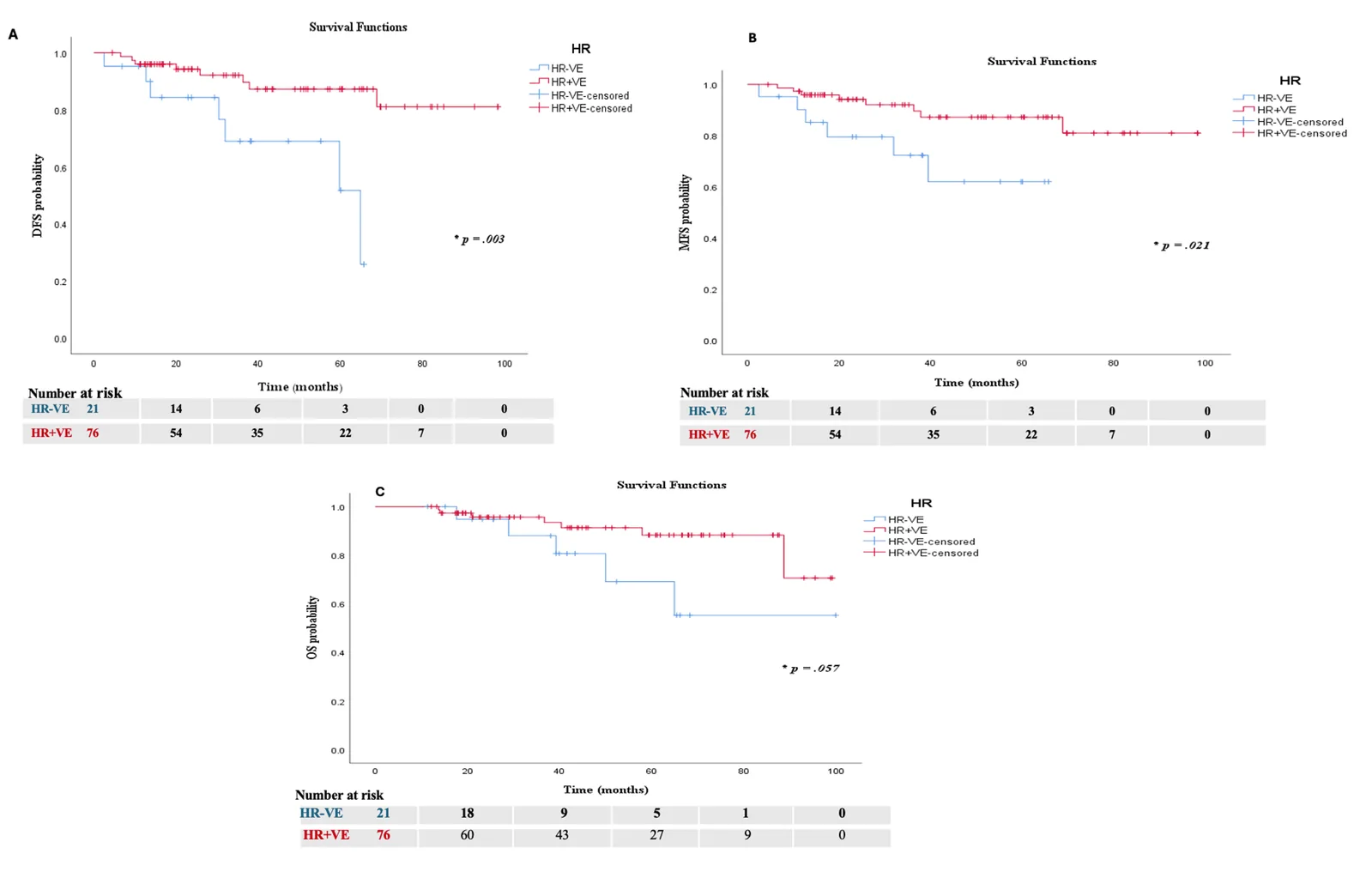
Survival outcomes of all patients based on HR status (A) Kaplan-Meier survival curve comparing DFS between HR+ (n=76) and HR- (n=21) groups. (B) Kaplan-Meier survival curve comparing MFS between HR+ (n=76) and HR- (n=21) groups. (C) Kaplan-Meier survival curve comparing OS between HR+ (n=76) and HR- (n=21) groups. p-values were calculated using the log-rank test. The number at risk is provided below the x-axis. DFS: disease-free survival, MFS: metastasis-free survival, OS: overall survival, HR: hormonal status

However, while the overall survival (OS) showed a numerical trend favoring the HR+ group, the difference did not reach statistical significance (p=0.057; Figure 2).

Survival rates stratified by HR and HER2 status 

Survival outcomes were further analyzed within each HER2 stratum to evaluate the impact of HR expression using Kaplan-Meier log-rank tests.

**Figure 3:**
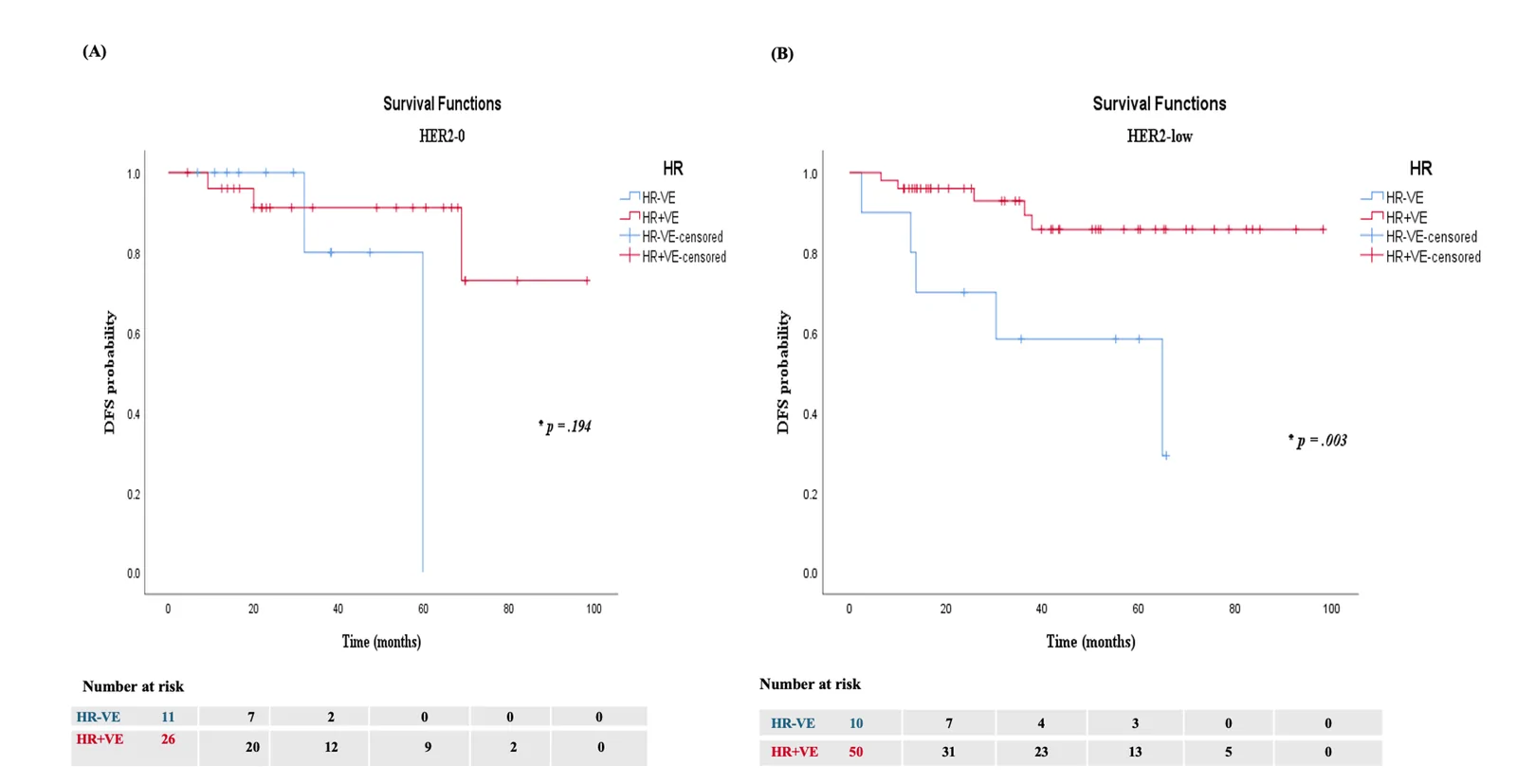
DFS rates of HER2-0 and HER2-low patients considering the HR status expression (A) Kaplan-Meier survival curve comparing DFS of HER2-0 patients between HR+ (n=26) and HR- (n=11) groups. (B) Kaplan-Meier survival curve comparing DFS of HER2-low patients between HR+ (n=50) and HR- (n=10) groups. p-values were calculated using the log-rank test for each stratum. The number at risk is provided below the x-axis. HER2: human epidermal growth factor receptor 2, HR: hormone receptor, DFS: disease-free survival

**Figure 4:**
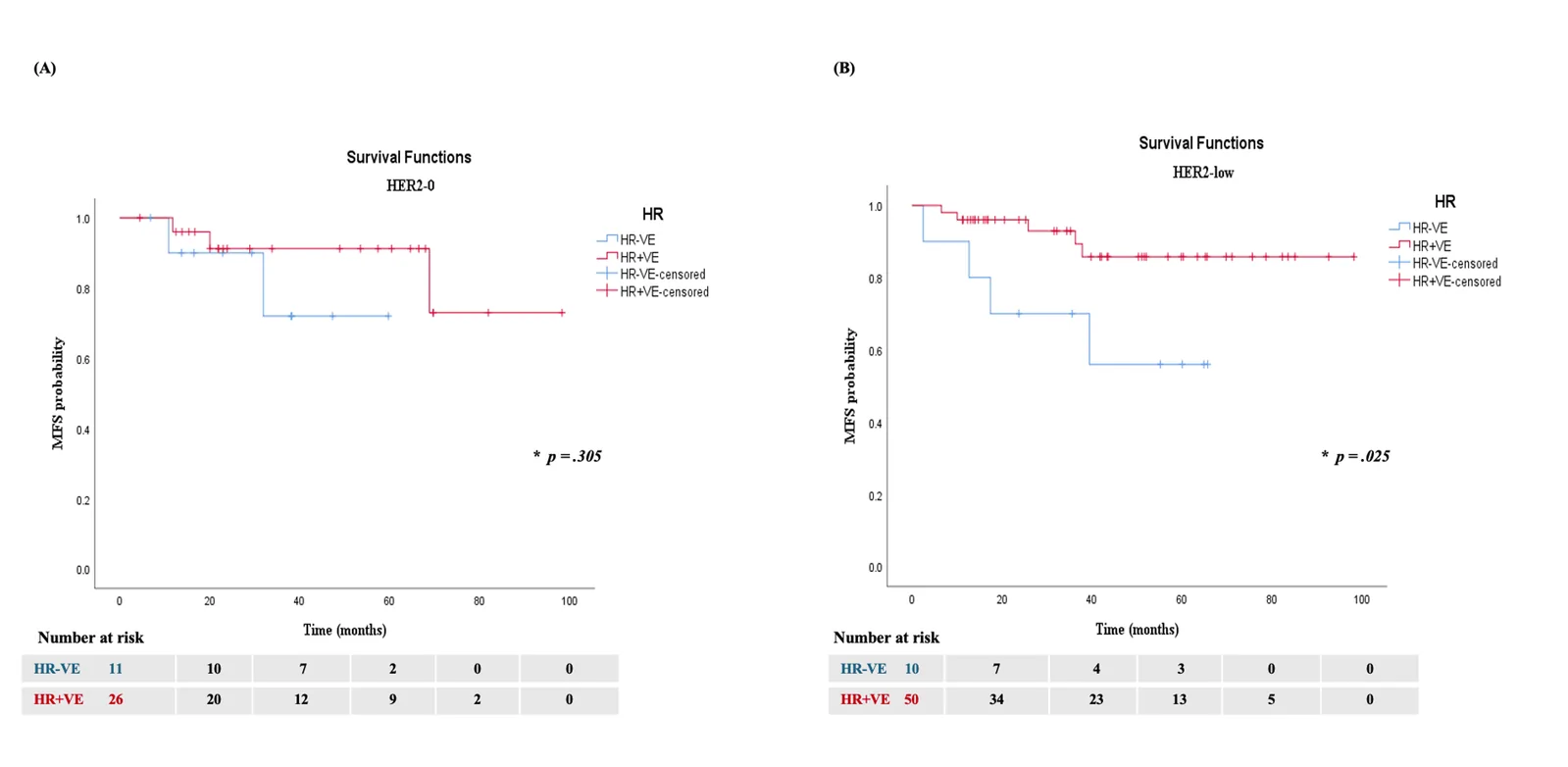
MFS rates of HER2-0 and HER2-low patients considering the HR status expression (A) Kaplan-Meier survival curve comparing MFS of HER2-0 patients between HR+ (n=26) and HR- (n=11) groups. (B) Kaplan-Meier survival curve comparing MFS of HER2-low patients between HR+ (n=50) and HR- (n=10) groups. p-values were calculated using the log-rank test for each stratum. The number at risk is provided below the x-axis. HER2: human epidermal growth factor receptor 2, HR: hormone receptor, MFS: metastasis-free survival

**Figure 5:**
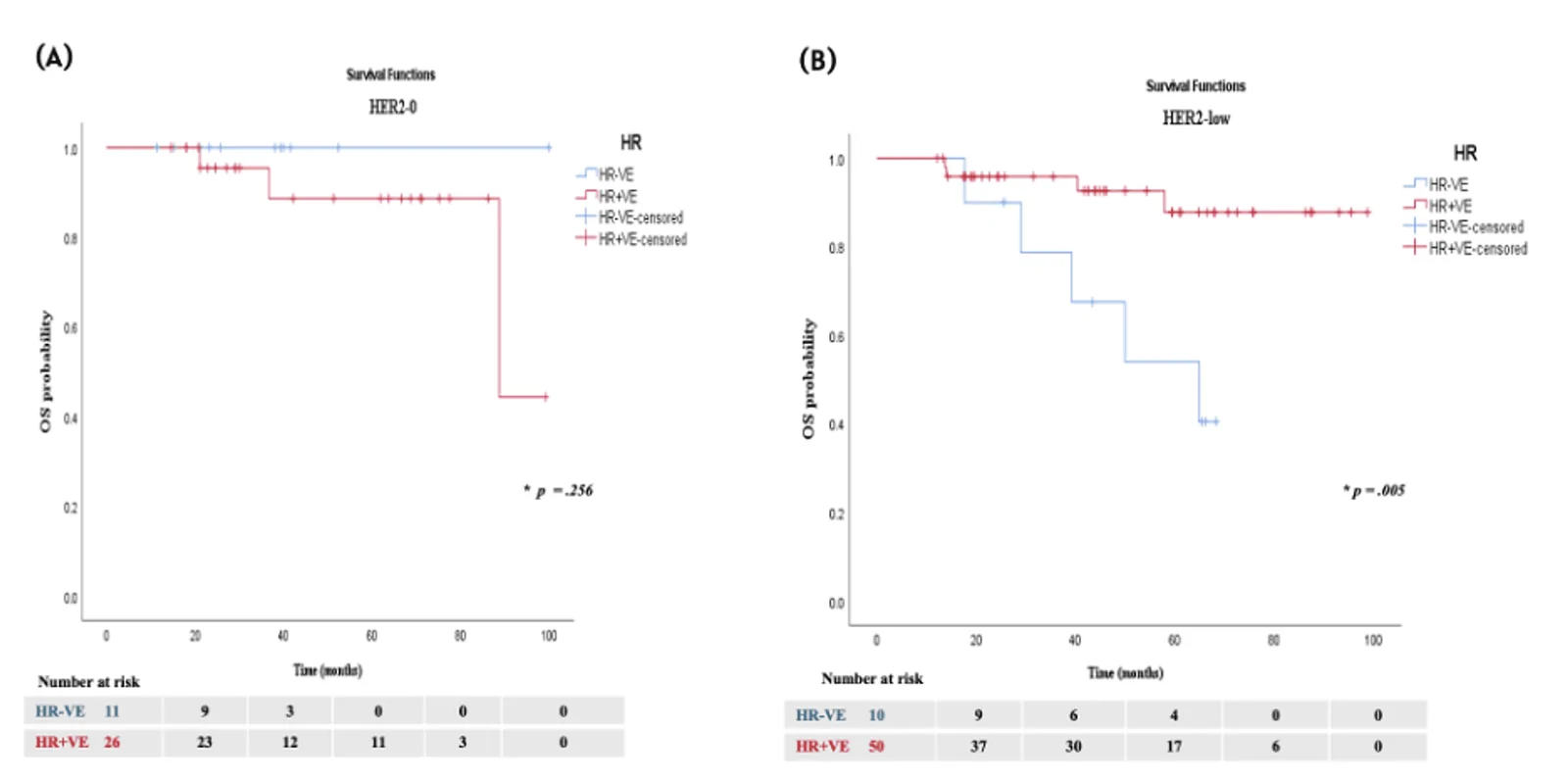
OS rates of HER2-0 and HER2-low patients considering the HR status expression (A) Kaplan-Meier survival curve comparing OS of HER2-0 patients between HR+ (n=26) and HR- (n=11) groups. (B) Kaplan-Meier survival curve comparing OS of HER2-low patients between HR+ (n=50) and HR- (n=10) groups. p-values were calculated using the log-rank test for each stratum. The number at risk is provided below the x-axis. HER2: human epidermal growth factor receptor 2, HR: hormone receptor, OS: overall survival

In the HER2-low cohort, patients with HR+ tumors demonstrated a marked survival advantage. The HR+/HER2-low subgroup achieved the highest survival rates, with a DFS and MFS of 87.99%. In contrast, the HR-/HER2-low subgroup showed significantly poorer outcomes, with DFS and MFS dropping to 44.5% and 45.57%, respectively. This survival advantage for HR+ patients was statistically significant across all end points (DFS: p=0.003 (Figure 3), OS: p=0.005 (Figure 4), MFS: p=0.025 (Figure 5)). 

In contrast, within the HER2-0 cohort, HR-positive patients also demonstrated numerically better survival outcomes than HR-negative patients; however, these differences did not reach statistical significance (DFS: p=0.194 (Figure 3), OS: p=0.256 (Figure 4), MFS: p=0.305 (Figure 5)).

These subgroup analyses suggest that the prognostic association of HR positivity was more evident within the HER2-low subgroup in this cohort. However, because the formal interaction analysis between HR status and HER2 status was not statistically significant (interaction p=0.325), these findings should not be interpreted as evidence that HER2 status modifies the prognostic effect of HR expression. Rather, they should be considered exploratory observations requiring confirmation in larger studies.

Univariate and multivariate analyses for disease-free survival (DFS)

The prognostic effect of clinicopathological variables on DFS was analyzed using Cox proportional hazards models (Table 5). In the multivariate analysis, HER2-low status did not emerge as an independent prognostic factor for DFS (p=0.354). Instead, Ki-67 expression > 20% (hazard ratio (HR): 8.27; p=0.032) and higher lymph node ratio (LNR) risk (HR: 7.67; p=0.019) were found as the main independent predictors of recurrence. Additionally, compared to no systemic treatment, neoadjuvant chemotherapy was linked with a significantly reduced risk of recurrence (HR: 0.078; p=0.041).

**Table 5:** Univariate analysis and multivariate analysis for disease-free survival Data presentation: Values are presented as HR with 95% CIs. Statistical analysis: Cox proportional hazards regression model was used for univariate and multivariate analysis. Statistical significance was set at p<0.05. HR: hazard ratio, CI: confidence interval, HER2: human epidermal growth factor receptor 2, HR: hormone receptor, LNR: lymph node ratio, LVI: lymphovascular invasion, Ref.: reference category

Variables	Univariate analysis (HR (95% CI))	Univariate p-value	Multivariate analysis (HR (95% CI))	Multivariate p-value
HER2 status, HER2-0 (Ref.) versus HER2-low	1.43 (0.44-4.58)	0.546	1.78 (0.52-6.12)	0.354
HR, HR- (Ref.) versus HR+	0.23 (0.08-0.66)	0.006	0.63 (0.15-2.69)	0.534
Ki-67 index, ≤20% (Ref.) versus >20%	3.99 (0.89-17.83)	0.070	8.27 (1.20-57.23)	0.032
Histological grade, low/intermediate (Ref.) versus high	2.80 (0.89-8.82)	0.078	2.44 (0.56-10.58)	0.233
LNR risk categories, low risk (Ref.) versus intermediate/high risk	3.22 (1.13-9.21)	0.028	7.67 (1.39-42.37)	0.019
LVI, LVI-negative (Ref.) versus LVI-positive	2.78 (0.87-8.87)	0.083	0.57 (0.10-3.36)	0.543
Clinical tumor size, T1-2 (Ref.) versus T3-4	3.35 (1.16-9.60)	0.024	3.28 (0.80-13.39)	0.097
Age (years), ≤50 (Ref.) versus >50	0.88 (0.31-2.53)	0.821	0.65 (0.18-2.41)	0.526
Chemotherapy timing, none (Ref.) versus systemic chemotherapy	1.00	0.640	1.00	0.116
Adjuvant versus none	0.51 (0.12-2.06)	0.350	0.11 (0.01-1.13)	0.063
Neoadjuvant versus none	0.59 (0.15-2.43)	0.473	0.08 (0.01-0.91)	0.041
Radiotherapy, no (Ref.) versus yes	0.96 (0.21-4.29)	0.965	0.28 (0.02-3.63)	0.330

In the univariate analysis, HR status showed a significant prognostic impact (p=0.006) with a strong protective effect (HR: 0.23). However, in the multivariate model, this effect was attenuated (p=0.534), showing that proliferation (Ki-67), LNR, and systemic therapy were more dominant factors influencing DFS in this study cohort.

To evaluate whether the prognostic impact of HER2-low status differed by hormone receptor (HR) expression, an interaction term (HR × HER2 status) was included in the Cox model. The interaction was not statistically significant (p=0.325), indicating that HER2-low status did not disproportionately affect disease-free survival (DFS) in either the HR-positive or HR-negative subgroups.

To further explore the biological relationship between HER2 expression and HR status, a subgroup analysis for DFS was conducted. Within the HER2-low subgroup, HR-positive status was associated with significantly improved DFS (HR: 0.184; 95% CI: 0.053-0.637; p=0.008). In contrast, while a similar protective trend was observed in the HER2-0 cohort, it did not reach statistical significance (HR: 0.282; 95% CI: 0.037-2.137; p=0.221) (Table 6).

**Table 6:** Univariate Cox regression of hormonal status variable for DFS stratified by HER2 status Data presentation: Data are presented as HR with 95% CIs. Statistical analysis: p-values were determined using univariate Cox proportional hazards regression analysis stratified by HER2 expression status. Statistical significance was defined as p<0.05. HR: hazard ratio, CI: confidence interval, HER2: human epidermal growth factor receptor 2, HR status: hormone receptor status, DFS: disease-free survival

Subgroup	Variable	HR	95% CI	p-value
HER2-low cohort	HR status (- versus +)	0.184	0.053-0.637	0.008
HER2-0 cohort	HR status (- versus +)	0.282	0.037-2.137	0.221

Although statistical significance was observed only within the HER2-low subgroup, the absence of a significant interaction indicates that these subgroup findings should not be interpreted as demonstrating a differential prognostic effect of HR according to HER2 status. Instead, they represent exploratory findings that may reflect limited statistical power, particularly within the small HR-negative subgroup, and warrant validation in larger prospective studies.

## Discussion

The findings of this research contribute to the global understanding of HER2-low breast cancer (BC) by providing real-world data from the Middle East. Our observed HER2-low prevalence of 61.9% (n=60/97) closely mirrors findings from substantial Western and Asian cohorts, such as those reported by Schettini et al. (≈55%--65%) [2] and other large datasets [8,23]. These consistently elevated rates across diverse populations challenge the traditional HER2-positive/negative dichotomy and support the emerging hypothesis that HER2 exists on a biological continuum rather than as a binary characteristic.

Consistent with existing research, our cohort was dominated by the hormone receptor-positive (HR+) subtype (n=76/97, 78.4%). Within the HR+ group, HER2-low expression was more prevalent than HER2-0 (n=50/76, 65.8% versus n=26/76, 34.2%). Notably, significantly more HER2-low patients were treated with adjuvant hormonal therapy compared to HER2-0 individuals (n=48/50, 96.0% versus n=22/26,84.6%; p=0.035). This is in accordance with observations by Cha et al., where HER2-0 tumors with lower HR expression often received less endocrine-based treatment and more chemotherapy [24].

Furthermore, our data revealed a higher incidence of invasive lobular carcinoma (ILC) in the HER2-low group (n=11/60, 18.3%) compared to the HER2-0 group (n=1/37, 2.7%), with borderline statistical significance (p=0.058). This is aligned with larger series reporting that between 33% and 65% of invasive lobular carcinomas (ILCs) were demonstrated to have HER2-low status [25-27].

Our comparison of HER2-low and HER2-0 tumors revealed similar clinical and pathological features, supporting the consensus that HER2-low status does not define a unique intrinsic subtype with inherent aggressiveness, consistent with most contemporary studies [28,29]. Earlier studies suggesting more favorable features in HER2-low tumors were later recognized as confounded by the disproportionate representation of hormone receptor-positive (luminal) tumors within the HER2-low category [30].

Our results reinforce this interpretation by revealing no intrinsic pathological benefit linked to HER2-low status itself. However, HR-stratified analysis revealed more nuanced differences. We observed a significant difference in clinical T-stage between the HR+/HER2-low and HR+/HER2-0 groups, with the HER2-0/HR+ subgroup encompassing all cT4 cases. Conversely, in the triple-negative (HR-) subgroup, HER2-low status was associated with higher lymphovascular invasion (LVI) rates (n=4/10, 40% versus n=1/11, 9.1%; p=0.035) and more aggressive pathological features, which is in line with observations from Korean, Japanese, and Chinese populations. Yang et al. [31], Zhang et al. [32], and Kang et al. [33] each reported that HR-/HER2-low tumors share molecular and phenotypic features with basal-like disease despite exhibiting low-level HER2 expression.

Our survival analysis showed no statistically significant differences in DFS, MFS, or OS between HER2-low and HER2-0 classifications when considered as a whole. However, in subgroup analysis, HR+/HER2-low patients achieved significantly higher survival rates across all end points compared to HR-/HER2-low patients (p<0.05 for all). Although similar trends were observed within the HER2-0 cohort, these differences did not reach statistical significance. Given the non-significant HR HER2 interaction test, this subgroup findings should be interpreted as exploratory rather than evidence that HER2 status modifies the prognostic effect of HR expression.

The multivariate and univariate analyses of our cohort showed that HER2-low status does not act as an independent prognostic marker for DFS. These results are in accordance with results from the French ESME registry, the German BRENDA registry, and meta-analyses by Schettini et al. [2], all of which concluded that HER2-low status lacks independent prognostic value in early-stage breast cancer [24,25,34,35], suggesting that HER2-low tumors behave similarly to HER2-0 tumors when adjusted for other factors. Instead, recurrence risk was dominated by biological proliferation (Ki-67) and the lymph node ratio (LNR). These findings reinforce the importance of traditional prognostic markers and high-quality systemic therapy in managing both HER2-low and HER2-0 disease. Across almost all breast cancer literature, nodal density (LNR) [36] and proliferation (Ki-67) [37] are consistently the “gold standards” for predicting recurrence.

To further explore the biological relationship between HER2 expression and hormone receptor (HR) status, we conducted a stratified univariate analysis to evaluate the prognostic impact of HER2-low status on DFS based on hormonal expression. In the HER2-low cohort, HR-positive status was a highly significant predictor of improved survival (HR: 0.184; 95% CI: 0.053-0.637; p=0.008). While a similar protective trend was observed in the HER2-0 cohort, it did not reach statistical significance (HR: 0.282; 95% CI: 0.037-2.137; p=0.325). Despite these divergent univariate p-values, the formal test for interaction between HER2 status and HR status was not statistically significant (p=0.325). These findings indicate that HR-positive status was associated with significantly better DFS within the HER2-low subgroup. However, because the formal interaction test was not statistically significant (p=0.325), there is insufficient statistical evidence to conclude that the prognostic effect of HR differs according to HER2 status. Therefore, these subgroup findings should be interpreted as exploratory observations requiring validation in larger cohorts.

Previous studies have suggested that luminal (HR+) and triple-negative (HR-) HER2-low tumors may differ biologically, whereas HER2-0 tumors may exhibit greater biological heterogeneity [38]. Although our findings are consistent with this hypothesis, the non-significant interaction test indicates that our data do not provide sufficient evidence to confirm that HER2 status modifies the prognostic effect of HR expression.

Our findings are consistent with previous studies demonstrating that hormone receptor status remains an important prognostic factor in HER2-low breast cancer [33,39]. However, our data do not demonstrate that HR has a different prognostic effect according to HER2 status. Despite a higher proportion of HR-positive patients expressed in HER2-low compared to patients with TNBC [40], an insignificant OS and DFS difference between the HER2-0 and HER2-low groups was reported, suggesting the possibility of HER2-low as an inferior prognostic factor of breast carcinomas at an early stage [41]. A meta-analysis study confirmed improved overall survival (OS) and disease-free survival (DFS) in HER2-low early breast cancer. That survival benefit appeared modulated by hormone receptor status. While the OS advantage was maintained in HR+ subgroups, it was attenuated or absent in triple-negative breast cancer (TNBC) [38].

While some large-scale studies in East Asian populations reported more favorable features in HER2-low tumors [42], our findings regarding high LVI rates (n=4/10, 40%) and poorer DFS in the TNBC/HER2-low subgroup align with emerging evidence suggesting that low-level HER2 expression in an HR-negative framework identifies a high-risk population with distinct metastatic potential requiring more intensive monitoring compared to their HER2-0 counterparts [40,43,44].

Our results also have regional significance. Considering the Middle East’s unique breast cancer epidemiology, characterized by earlier diagnosis and different subtype distributions, the prevalence of HER2-low, clinicopathological features, and survival rates within our group closely mirrors global statistics. While HER2-low is not “prognostic” (predicting survival), it remains “predictive” (predicting response to new ADCs like trastuzumab deruxtecan). This suggests that novel ADC-based therapeutic approaches could be broadly relevant to Middle Eastern populations. Furthermore, it illustrates the importance of enhanced HER2 scoring consistency, especially in light of the persistent challenges in distinguishing IHC 0 from 1+, a differentiation that directly influences ADC eligibility [45,46].

Strengths and limitations

The primary strength of this study includes using the lymph node ratio (LNR), which provides a superior assessment of total nodal burden compared to conventional N-staging and absolute positive node counts for predicting recurrence and survival [47,48]. Several limitations must be acknowledged. First, the retrospective design from a single tertiary center introduces potential selection bias and lacks external cohort validation. Second, the sample size, particularly within the HR−/HER2-low subgroup, limits statistical power for subgroup survival analysis and formal interaction testing, yielding wide confidence intervals for high-risk subsets (such as elevated Ki-67 and LNR) and necessitating that these subgroup findings be regarded as exploratory and hypothesis-generating. Third, the mean follow-up duration of 45.5 months may be insufficient to capture late recurrences typical of hormone receptor-positive disease. Finally, while HER2 scoring was strengthened by routine dual-pathologist review, formal interobserver reproducibility metrics (kappa statistics) were not calculated. Despite these constraints, which are common across early HER2-low literature, the alignment of our primary findings with multiple large-scale global datasets reinforces their clinical validity.

## Conclusions

This retrospective analysis corroborates the high prevalence and clinical value of HER2-low breast cancer. Although HER2-low status was not identified as an independent prognostic factor, HR-positive patients demonstrated favorable survival outcomes, particularly within the HER2-low subgroup. However, given the non-significant interaction test, these subgroup findings should be interpreted as exploratory rather than indicative of a distinct interaction. HER2-low currently remains primarily a predictive biomarker for HER2-targeted antibody-drug conjugates (ADCs) rather than an independent prognostic marker.
